# Correlation between pleth variability index and ultrasonic inferior vena cava-collapsibility index in parturients with twin pregnancies undergoing cesarean section under spinal anesthesia

**DOI:** 10.1186/s40001-022-00771-3

**Published:** 2022-08-06

**Authors:** Huiying Zhang, Hongmei Yuan, Huiling Yu, Yue Zhang, Shanwu Feng

**Affiliations:** 1 Department of Anesthesiology, Women’s Hospital of Nanjing Medical University, Nanjing Maternity and Child Health Care Hospital, No. 123 Tianfei Xiang, Mochou Road, Jiangsu 210004 Nanjing, People’s Republic of China; 2 Department of Anesthesiology, Women’s Hospital of Nanjing Medical University, Nanjing Maternity and Child Health Care Hospital, No. 123 Tianfei Xiang, Mochou Road, Nanjing, Jiangsu 210004 People’s Republic of China

**Keywords:** Pleth variability index, Twin pregnancies, Inferior vena cava-collapsibility index, Spinal anesthesia

## Abstract

**Background:**

To explore the correlation and consistency of non-invasive pleth variability index (PVI) combined with ultrasonic measurement of inferior vena cava-collapsibility index (IVC-CI) in parturients with twin pregnancies undergoing cesarean section under spinal anesthesia.

**Methods:**

Forty-seven twin pregnancies women undergoing elective cesarean section were selected. The ASA score was rated as I–II, aged from 18 to 45 years. Spinal anesthesia was performed at L3–4. PVI and IVC-CI, general data (BMI, gestational weeks, operation duration, blood loss), MAP, temperature sensory block level and adverse reactions were recorded at baseline (T1) and completion of testing the level of spinal anesthesia (T2).

**Results:**

The correlation coefficient analysis of baseline IVC-CI% and PVI revealed that the Pearson's coefficient was 0.927, > 0.4. Thus, pre-anesthesia IVC-CI% had a strong correlation with PVI, with *R*^*2*^ of 85.69%. The correlation coefficient analysis of post-anesthesia IVC-CI% and PVI revealed that the Pearson's coefficient was 0.904, > 0.4. Thus, post-anesthesia IVC-CI% had a strong correlation with PVI, with *R*^*2*^ of 81.26%.

**Conclusion:**

PVI is strongly consistent with ultrasound measurement of IVC-CI twin pregnancies, which can be used as a valuable index for predicting the volume in parturients with twin pregnancies undergoing cesarean section under spinal anesthesia.

*Trial registration* This study was registered on ClinicalTrials.gov with clinical trial registration number of ChiCTR2200055364 (08/01/2022).

## Keypoints

- The combination of pleth variability index (PVI) and inferior vena cava-collapsibility index (IVC-CI) has correlation and consistency in cesarean delivery in twin pregnancies.
- The distribution intervals of IVC-CI% and PVI were relatively widely distributed after anesthesia when the blocking plane was in the T5 stratum, narrower at T6, and intermediate at T7.
- PVI is a valuable predictor of cesarean delivery volume in twin pregnancies in clinical practice.

## Introduction

As systemic circulating blood volume increases by 40–50% during pregnancy compared to non-pregnancy, especially in twin pregnancies, which are more prone to volume-related perinatal complications than singleton pregnancy, and consequently to cardiovascular and cerebrovascular events, better volume monitoring is in line with the concept of enhanced recovery after surgery (ERAS) [1, 2]. A non-invasive hemoglobin monitor (Radical 7, Masimo^®^, USA) can measure the pleth variability index (PVI) non-invasively, and the PVI is accurate in assessing volume status [3]. Goal-directed fluid therapy (GDFT) optimizes intraoperative tissue perfusion [3, 4]. Monitoring of inferior vena cava (IVC) diameter and respiratory variability is often used to assess fluid responsiveness and volume loading status. The combination of PVI with inferior vena cava-collapsibility index (IVC-CI) provides improved sensitivity and enhanced specificity, making it a valuable clinical predictor of maternal volume in twin pregnancies [5]. There is a paucity of systematic studies on the use of perinatal volume assessment tools to accelerate the surgical recovery of women undergoing cesarean section and on the management of volume in twin pregnancies [5, 6]. Given the advantages of non-invasive and continuous PVI, combined with the role of IVC-CI in volume prediction, this trial was conducted to investigate the correlation and concordance between non-invasive PVI and ultrasound-guided IVC-CI in cesarean delivery in twin pregnancies.

## General information

This study was registered with the Chinese Clinical Trials Registry (ChiCTR2200055364), and approved by the Medical Ethics Committee of Women's Hospital of Nanjing Medical University (Nanjing Maternity and Child Health Care Hospital) (No. 2021-0729), and all mothers signed an informed consent form. Forty-seven elective termed twin pregnancies cesarean deliveries between January 2022 and March 2022 were selected, with American Society of Anesthetists (ASA) score of I–II, aged 18–45 years. Exclusion criteria: (1) women with local skin infection or ulcer in the subxiphoid process or left fingers; (2) women with a history of allergy to ultrasound couplers; (3) women with pre-eclampsia, gestational diabetes mellitus or peripheral nerve dysfunction; (4) women with absolute contraindications to intraspinal anesthesia; (5) women who are participating in other clinical trials or have participated in other clinical trials within 3 months prior to enrollment; and (6) women who, in the judgment of the anesthetist in charge of the trial, may be at increased risk or may not have adequate data from the trial. After subarachnoid administration, some patients may receive vasoactive drugs due to hypotension, which may affect the results of the study. To eliminate this effect, all cases of transient hypotension were excluded in this study. All parturients with dominant hypotension after intravascular anesthesia have been excluded from this trial.

## Study design

### Anesthetic protocol

No preoperative medication was given to any of the 47 women with twin pregnancies undergoing elective cesarean section, and the operating room temperature was maintained at 22 °C. The right side of the mother’s buttock was padded so that she was in a supine position with a left tilt of 15°. HR, SBP, DBP, MAP, SpO_2_ and ECG were monitored by an anesthetist and PVI was measured by an anesthetic nurse with a Masimo Radical 7 probe attached to the tip of the index finger or other finger of the woman's left hand, without the knowledge of the anesthetist. The left upper limb vein is opened without pre-dilation. Infusion of 500 ml sodium lactate Ringer’s solution at a rate of 0.2 m1/kg/min, followed by a continued infusion of 130/0.4 hydroxyethyl starch at the same rate until the end of the procedure. Spinal anesthesia was performed in the left lateral position with the puncture site be L3-4. Ultrasound scans were performed prior to epidural anesthesia by portable color Doppler ultrasound equipped with a 2–5 MHz convex array probe (SonoScape Medical Corp., Shenzhen, China). Ultrasound imaging was performed with the patient in the left lateral recumbent position and an epidural needle was placed in the same position. Briefly, the ultrasound probe was placed in the paramedian sagittal oblique plane, counting down from the 12th rib or up from the sacrum, and both ultrasound scanning methods were used to identify the intervertebral space. A 16G needle was used first to locate the epidural cavity, then a 25G lumbar puncture needle was used to puncture through the inner lumen of the epidural needle into the subarachnoid space. After cerebrospinal fluid outflow was observed, heavy gravity 10% GS (1 ml) and 0.75% ropivacaine (Nelapine^®^, AstraZeneca) 15 mg (2 ml) of a mixture of 3 ml was injected into the subarachnoid space, the injection speed was 10 s per milliliter. After completing the local anesthetics, the woman was returned to a left-leaning 15° supine position and inhaled oxygen through a nasal cannula at 2 l/min. The temperature sensory block plane was tested with an alcohol swab and the operation was started when the block plane reached the T7 level and above. If the block plane was insufficient within 10 min, the patient was excluded from the study. The amount of blood loss during cesarean section was estimated by gauze weighing method combined with standard attractor quantitative method. The gauze to be used in the operation was weighed and recorded with a fixed electronic scale preoperatively, and the gauze soaked by blood was weighed postoperatively, and the subtractive difference was calculated as 1 g (blood weight) = 1 ml (blood volume). In addition, the total amount of blood in the standard aspirator was recorded, while amniotic fluid is collected in another separate suction device after rupture of membrane. When gauze weighing and standard attractor quantitative are added together, the amount of bleeding is estimated.

### IVC-CI measurements under ultrasound (US)

The woman was placed in the supine position with a left tilt of 15°. The anesthetist took a long-axis subxiphoid view with a phased-array probe under the right rib cage and probed the inferior vena cava longitudinally, 2 cm near the entrance to the right atrium in the M mode. Valsalva respiration was used to monitor the maximum (Dmax) and minimum (Dmin) value of inferior vena cava variation with respiration during a complete cycle of spontaneous breathing. The Dmax and the Dmin were repeated 3 times and averaged to 1 decimal place.

## Observation items and indicators

A non-invasive blood pressure cuff was placed on the right upper arm and PVI monitoring (Pulse CO-Oximeter^®^ continuous measurement, software version V 1.1.1.3, using a SET^®^ sensor) was performed with a non-invasive hemoglobin meter (Radical 7, Masimo^®^, USA) with the probe placed on the fingertip of left hand, and PVI monitoring was continued after admission to the operating room until the end of the operation. Measurements of IVC-CI under US were taken at 2 time points after admission to the operating room, prior to anesthesia in a relatively stable sympathetic state (T1) and after completion of testing the level of intravertebral anesthesia (T2):

1. Record maternal body mass index (BMI), gestational week and comorbidities.
2. Record the time of the operation and the amount of bleeding during the cesarean section.
3. Record maternal MAP, HR, and other hemodynamic parameters at T1, T2.
4. Record the maternal thermo-sensory blocking plane after intravertebral anesthesia (alcohol-impregnated cotton swab method). The individual who made the sensory level assessments was unaware of the trial subgroup.
5. Measure and record of IVCmax and IVCmin at T1 and T2 and the PVI values at the corresponding time points. The measurement of IVC-CI was carried out by a graduate doctor who was also unaware of the trial subgroup.
6. To record adverse reactions such as ischemia at the fingertip and allergy to the ultrasound coupling agent at the end of the procedure.

## Statistical analysis

A sample size was selected for estimation using G-power software (v.3.1.9.2) with a two-tailed α of 0.05 and an efficacy (1-β) of 90%, requiring a minimum of 47 parturients. The sample size was chosen to detect a medium effect size (0.48) with 90% actual power using a two-tailed paired *t*-test.

Data normality was assessed using the Anderson–Darling test, and normally distributed measures were expressed as mean ± standard deviation ($\bar{x}$ ± s), while median (M) and interquartile range (IQR) were presented. The data were first tested for outliers using Grubbs’ test, *P* < 0.05, as the data had outliers and needed to be pre-processed. Pearson correlation analysis was then used to confirm whether there was a correlation based on the Pearson coefficient *r*. Finally, regression analysis was used to determine the utility of the regression variance based on *R*^*2*^ and *P*. When the sample size exceeds 9, if the absolute value of the correlation coefficient reaches 0.7, the two variables were considered to be correlated; when the sample size exceeds 25, if the absolute value of the correlation coefficient reaches 0.4, the two variables were considered to be correlated. All the data were downloaded from the Pulse CO-Oximeter^®^ using software (v.1.1.1.3, Radical 7, Masimo^®^, USA) and analyzed with Minitab^®^ (v.21.1, 64-bit).

## Results

A total of 47 women with twin pregnancies were included in this study, with successful surgery and satisfactory anesthesia (Table 1).

**Table 1:** General information (*n* = 47)

Variable	Mean	SD
BMI (kg/m^2^)	29.00	3.13
SBP (mmHg)	130.66	9.51
DBP (mmHg)	74.32	8.09
MAP (mmHg)	93.10	7.39
Operation time (min)	54.87	8.05
Surgical bleeding volume (ml)	486.80	96.20

### Pre-anesthesia and post-anesthesia correlation analysis between IVC-CI% and PVI

#### Correlation analysis between IVC-CI% and PVI before anesthesia

**Fig. 1:**
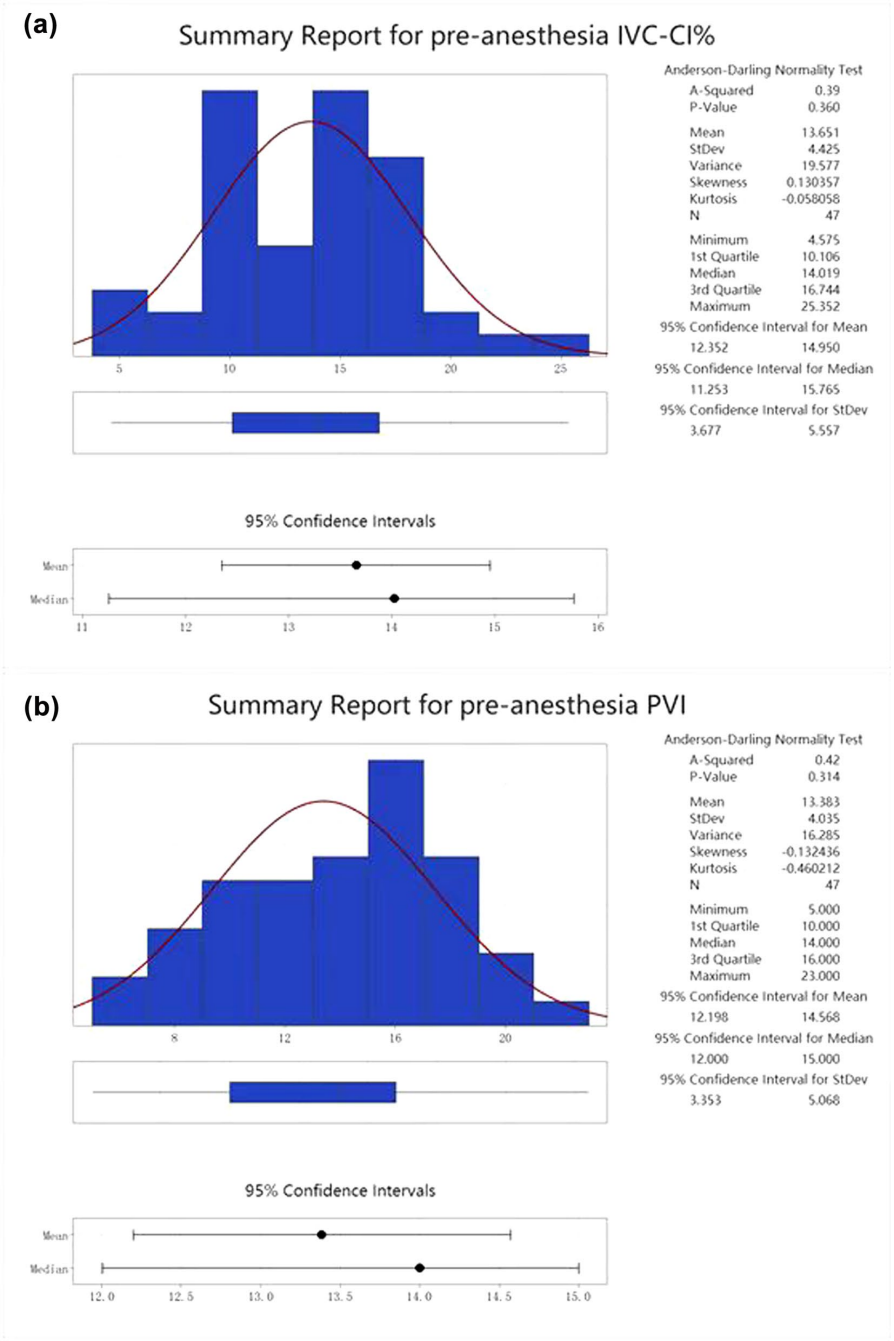
**a** Basic information on pre-anesthesia IVC-CI% data. **b** Basic information on pre-anesthesia PVI data

The basic information of IVC-CI% was mean 13.651, 95% CI of mean [12.352,14.950]; standard deviation 4.425; median 14.019 [10.106,16.744]; the normal distribution of the data checked *P* was 0.360, indicating that the data were normally distributed (Fig. 1a). The basic information of PVI was mean 13.383, with a 95% CI of [12.198,14.568] for the mean, a standard deviation of 4.035 and a median of 14.00 [10.000,16.000]; *P* for the normal distribution of the data checks out at 0.314, indicating that the data are normally distributed (Fig. 1b).

**Fig. 2:**
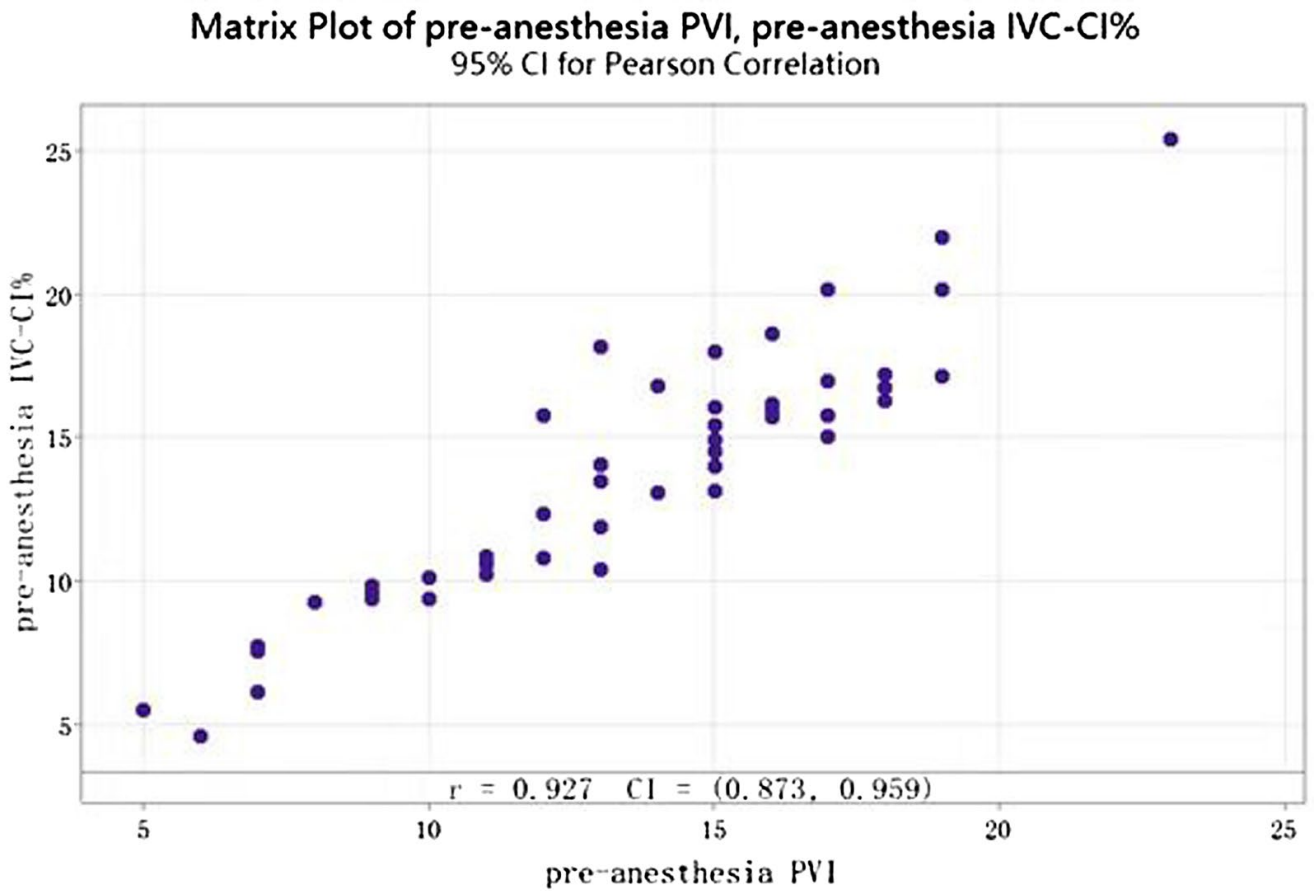
Correlation analysis of pre-anesthesia IVC-CI% and PVI

Analysis of the correlation coefficient between pre-anesthesia IVC-CI% and PVI revealed that the Pearson coefficient was 0.927, > 0.4, and pre-anesthesia IVC-CI% was strongly correlated with PVI (Fig. 2).

#### Correlation analysis between IVC-CI% and PVI after anesthesia

**Fig. 3:**
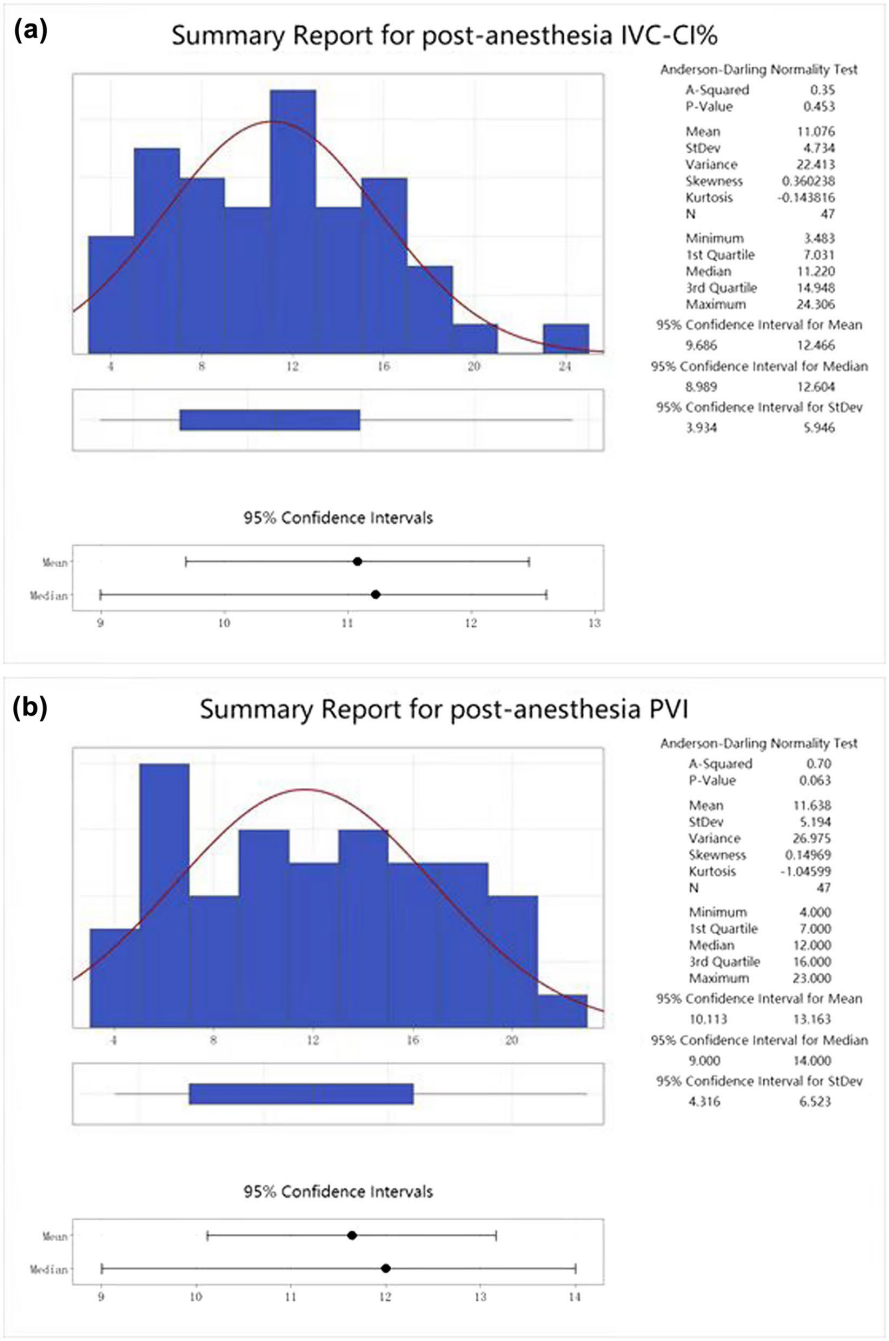
**a** Basic information on post-anesthesia IVC-CI% data. **b** Basic information on post-anesthesia PVI data

The basic information of IVC-CI% was mean 11.076; 95% CI of mean [9.686,12.466]; standard deviation 4.734; median 11.220 [7.031,14.948]; the normal distribution of the data was checked with *P* of 0.453, indicating that the data were normally distributed (Fig. 3a). Basic information of PVI was mean 11.638; 95% CI of the mean was [10.113,13.163] with a standard deviation of 5.194; median 12.00 [7.000,16.000]; the normal distribution of the data checked *P* was 0.063, indicating that the data were normally distributed (Fig. 3b).

**Fig. 4:**
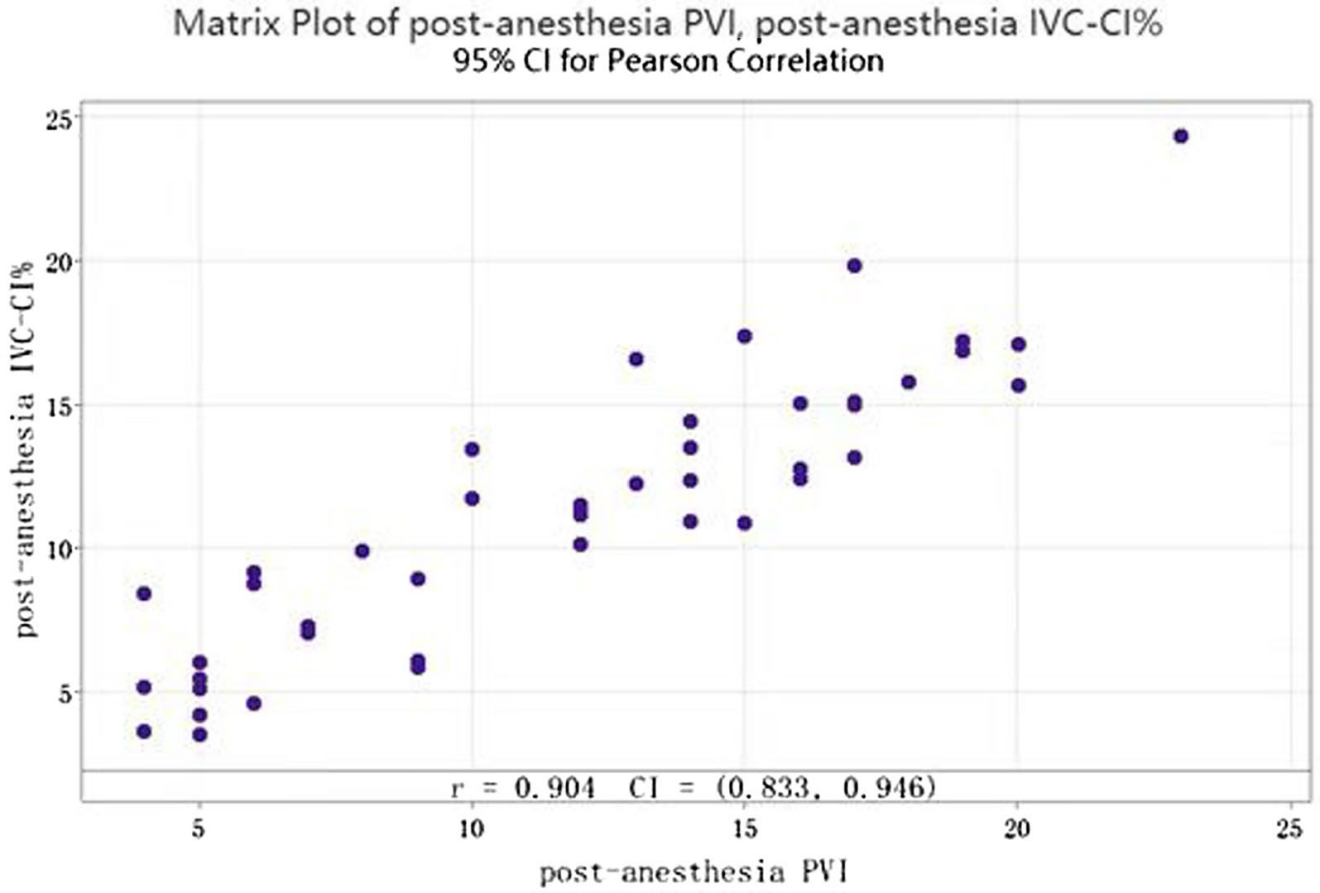
Correlation analysis of post-anesthesia IVC-CI% and PVI

Correlation coefficient analysis of post-anesthesia IVC-CI% and PVI revealed that the Pearson coefficient was 0.904, > 0.4, and post-anesthesia IVC-CI% was strongly correlated with PVI (Fig. 4).

### Regression analysis

#### IVC-CI% before anesthesia versus PVI before anesthesia

**Fig. 5:**
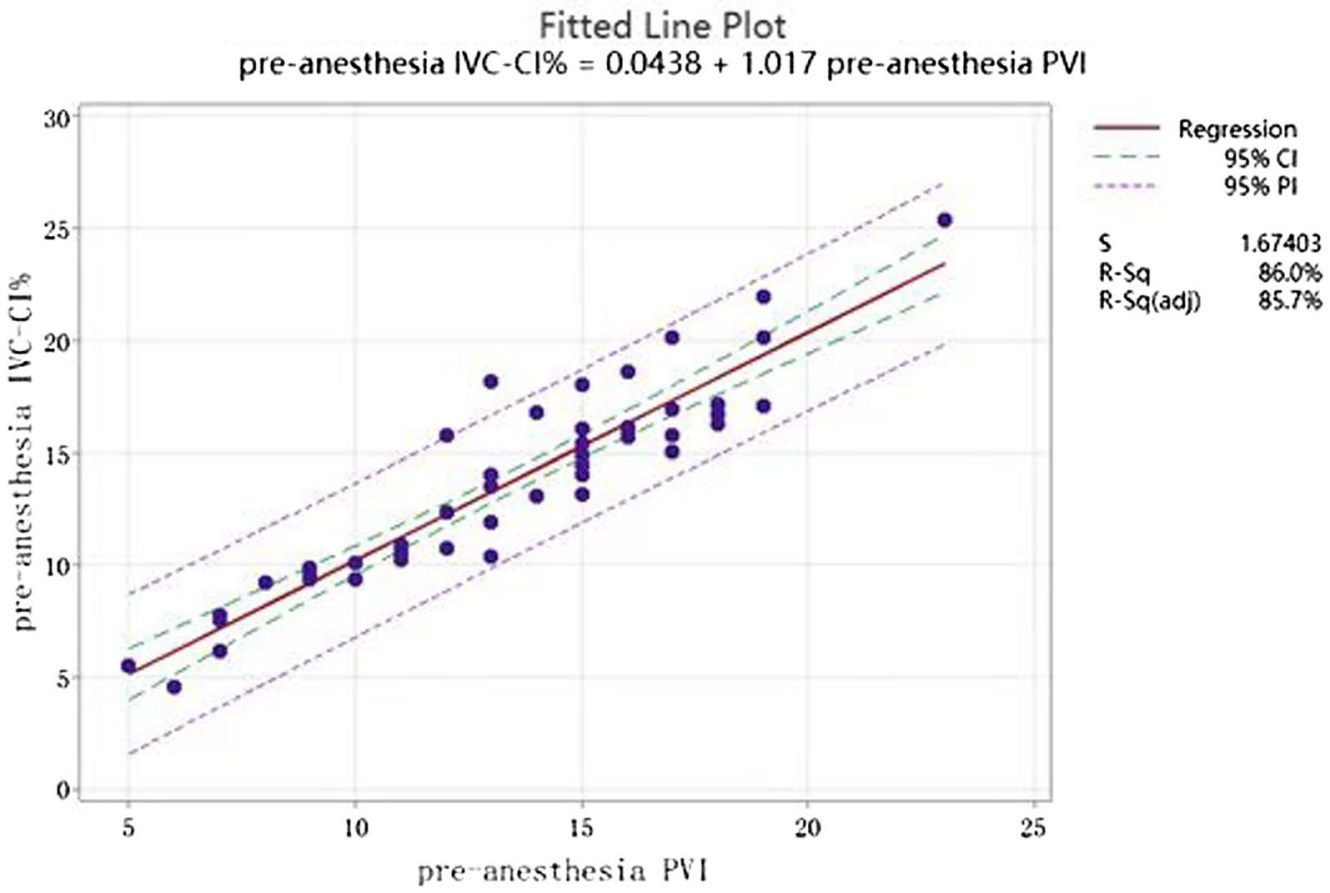
Fitted pre-anesthesia PVI to IVC-CI% regression equation. Pre-anesthesia IVC-CI% = 0.044 + 1.0168 pre-anesthesia PVI

The above analysis revealed that the correlation coefficient between IVC-CI% before anesthesia and PVI before anesthesia was 0.927, which is a strong correlation, and using regression analysis, the regression equation of the two could be derived, i.e., the value of IVC-CI% before anesthesia was estimated from the value of PVI before anesthesia, with *R*^*2*^ of 85.69% (Fig. 5).

#### Regression analysis: IVC-CI% after anesthesia versus PVI after anesthesia

**Fig. 6:**
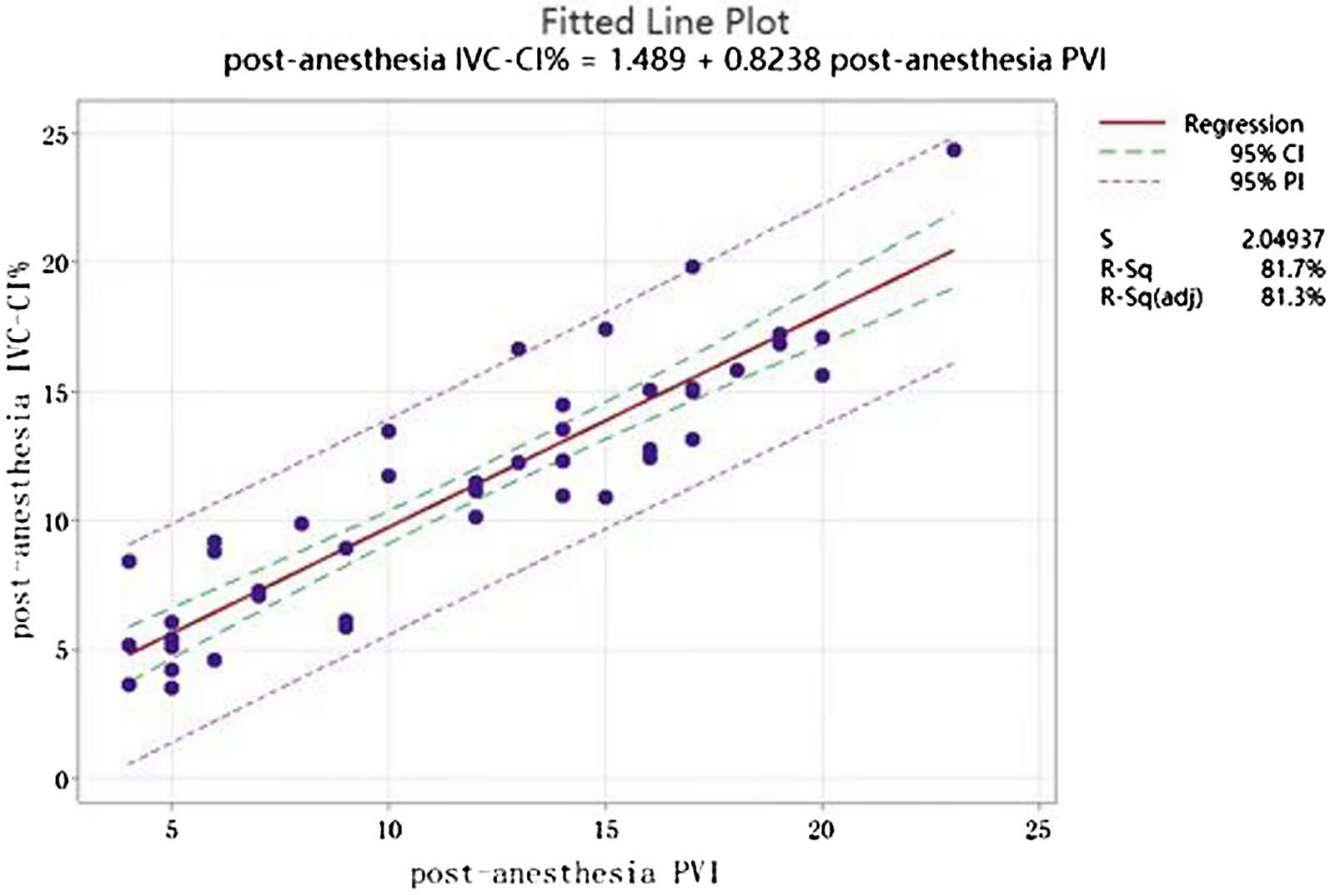
Fitted post-anesthesia PVI to IVC-CI% regression equation. Post-anesthesia IVC-CI% = 1.489 + 0.8238 post-anesthesia PVI

The above analysis revealed that the correlation coefficient between IVC-CI% before anesthesia and PVI before anesthesia was 0.904, which is a strong correlation, and using the regression analysis method, the regression equation of the two could be derived, i.e., the value of IVC-CI% before anesthesia was estimated from the value of PVI before anesthesia, with *R*^*2*^ of 81.26% (Fig. 6).

### Interval plot findings of IVC-CI%, PVI and temperature sensory block plane before and after anesthesia

**Fig. 7:**
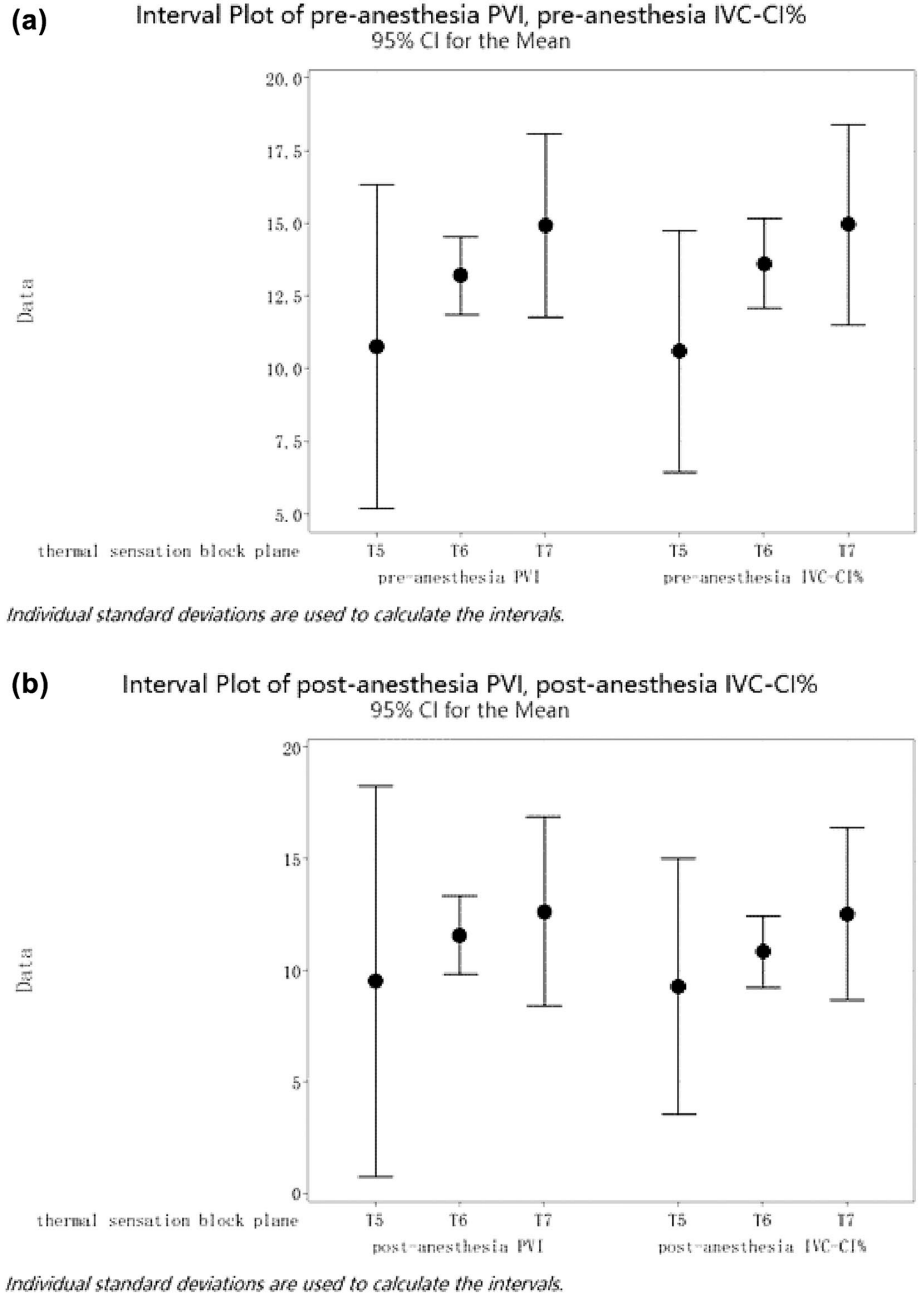
**a** 95% CI for per-anesthesia IVC-CI% and PVI at different thermal sensation block planes. **b** 95% CI for post-anesthesia IVC-CI% and PVI at different thermal sensation block planes

The high and low temperature sensory block planes were correlated with volume redistribution. After stratified analysis of the temperature sensory block planes T5/T6/T7, it was found that IVC-CI% and PVI had the same distribution interval before and after anesthesia (Fig. 7a and b). With the blocking plane at the T5 level, the distribution intervals of IVC-CI% and PVI were relatively widely distributed after anesthesia, and at T6, the distribution intervals were narrower, with T7 in the middle (Fig. 7a, b).

## Discussion

In this study, the correlation coefficient between IVC-CI% before anesthesia and PVI was found to be 0.927, > 0.4, with a strong correlation between IVC-CI% before anesthesia and PVI, with *R*^2^ of 85.69%; the correlation coefficient between IVC-CI% after anesthesia and PVI was found to be 0.904, > 0.4, with a strong correlation between IVC-CI% after anesthesia and PVI. IVC-CI% had a strong correlation with PVI, with *R*^2^ of 81.26%. In addition, the temperature sensory block planes were correlated with volume redistribution, and a stratified analysis of temperature sensory block planes T5/T6/T7 showed that IVC-CI% had the same distribution interval as PVI before and after anesthesia. The distribution intervals of IVC-CI% and PVI were relatively widely distributed after anesthesia when the blocking plane was in the T5 stratum, narrower at T6, and intermediate at T7.

The non-invasive hemoglobin meter (Radical 7, Masimo®, USA) is capable of measuring hemoglobin concentration (SpHb), pulse oxygen perfusion index (PI) and pleth variability index (PVI) non-invasively and continuously [7]. Some studies have shown that PVI has a certain accuracy in evaluating capacity status, which is similar to the stroke volume variation (SVV) [8]. PVI reflects the respiratory variability of pulse volume and is very sensitive to changes in preload. Gagné et al. have shown that a larger basal PVI value before anesthesia is associated with hypotension after cesarean spinal anesthesia with an AUC of 0.87 [9]. Especially in twin pregnancies, the increased blood volume at the time of labor and the significant blood pressure fluctuations may cause ischemia in vital organs and even postoperative cognitive dysfunction (POCD) and perioperative cardiovascular and cerebrovascular events. PVI-guided volume therapy is goal-directed fluid therapy (GDFT), and GDFT is an individualized infusion protocol to maintain ideal volume status, which can shorten hospital days, reduce complications, and improve postoperative regression. PVI monitoring is based on a probe that senses cyclic changes in tissue blood flow due to cardiac pulsation based on the principle of light absorption. In the measurement of pulse oximetry, tissues such as skin and non-pulsatile venous blood flow absorb a constant dose of light, known as quantitative absorption, while pulsatile arterial blood flow absorbs a variable dose of light, known as variable absorption, and the ratio of variable absorption to quantitative absorption is PI [7–9]. The magnitude of change in PI during a respiratory cycle is known as PVI, which is calculated as PVI = (Pimax − PImin)/PImax × 100% [7, 8]. An increase in PVI indicates hypovolemia, and a decrease in PVI after fluid resuscitation indicates that hypovolemia is improving. Therefore, PVI-guided GDFT can effectively reduce blood lactate level compared to conventional methods of volume therapy, which can more significantly improve tissue perfusion and oxygen supply and better maintain perioperative hemodynamic stability, thus optimizing intraoperative tissue perfusion, dynamically monitoring blood supply to vital organs, reducing blood pressure fluctuations, and reducing perinatal complications such as POCD.

The advantages of US as a visualization technique include safety, non-radiation and portability, and its comprehensive assessment, airway management, stress management, pain management, prognosis optimization and other perioperative management roles to accelerate maternal surgical recovery. Among the US assessments of circulatory function, the most widely available is the monitoring of IVC diameter and respiratory variability to assess fluid responsiveness and volume load status [10]. The principle is that during mechanical ventilation or spontaneous breathing, intra-thoracic pressure changes periodically and the IVC return resistance changes periodically with it, when the circulating volume is insufficient, the more the IVC return blood flow is affected by intra-thoracic pressure changes, the more pronounced its respiratory variability. The IVC is influenced by intra-thoracic respiratory cycle pressure changes, such as venous return factors, pulmonary circulation resistance, and right heart preload, but the specific reference standards for IVC width and respiratory variability are still not uniform [11]. When maternal blood volume is insufficient, IVC collapse is significantly greater; when blood volume is excessive, the IVC is relatively full. Due to its highly compliant nature, the IVC collapses during inspiration and expands during expiration. According to the formula for calculating the IVC-CI, IVC-CI = [(Dmax − Dmin/Dmax)] × 100%. It has been shown that the IVC collapse index decreases with increasing weeks of gestation [(40.8 ± 15.2)% at < 14 weeks of gestation, (32.6 ± 19.9)% at 20–28 weeks, and (26.5 ± 11.8%) at 28–34 weeks], with a rebound [(32.2 ± 19.9)%] by full term (> 27 weeks), with a significant difference (*F* = 3.557, *P* = 0.017), with < 14 weeks of gestation significantly higher than 28–34 weeks (*P* = 0.009) [11, 12]. PVI is effective in determining changes in blood volume after general anesthesia combined with epidural block and has recently been used successfully in some studies to assess volume status in spontaneously breathing women [11, 12]. The limitations of this study may lie in the fact that intraoperative PVI does not reflect maternal volume status because of the unstable tidal volume and surgical maneuvers during spontaneous breathing. The Masimo^®^ Radical 7 (SET^®^ sensor) used in this study is more stable than the Masimo^®^ Radical 5 used in other studies, minimizing interference from autonomic respiratory variability. We believe that the combination of the PVI with the US measurement of the IVC-CI provides better sensitivity and enhanced specificity as a clinically valuable predictor of maternal volume in twin pregnancies [11, 12]. Previous editions of the relevant guidelines recommended maternal measurement in the left lateral position and at end-expiration, with the latest version recommending the supine position, but due to the greater impact of the enlarged uterus on IVC reflux compression in the supine position in women with twin pregnancies, we have adopted a 15° left lateral position to reduce the impact on IVC [13].

Blood volume begins to increase at 6–8 weeks of gestation, peaking at 32–34 weeks. Systemic circulating blood volume increases by 40–50% during pregnancy compared to non-pregnancy, and systemic vascular resistance decreases, triggering huge physiological changes in the hemodynamic balance during pregnancy, and the enlarged uterus compresses the IVC in late pregnancy, leading to a further decrease in return blood volume [13]. Volume monitoring and maintenance of circulatory stability are therefore particularly important, with a high incidence of hypotension following cesarean section anesthesia, combined with preoperative fasting and gestational hypertension, resulting in maternal relative volume deficit. In particular, twin pregnancies belong to high-risk pregnancy, with up to 40% of twin pregnancies likely to be associated with gestational hypertension, three to four times more than singleton pregnancy, and are more likely than singleton pregnancy to have volume-related perinatal complications, such as gestational hypertension, pulmonary edema and abnormal postpartum hemorrhage; they are also closely associated with a variety of neonatal complications, such as neonatal asphyxia, and neonatal respiratory distress syndrome [14]. It is extremely important to establish a real-time, objective, accurate, effective, and bedside operable method of perinatal blood volume monitoring in twin pregnancies. Although central venous pressure (CVP) and continuous cardiac output (PiCCO) are currently available for circulating volume assessment, they are invasive and have many associated complications and contraindications, limiting their use in the perinatal period. Clinical guidelines recommend the optimal delivery of a dichorionic diamniotic twins (DCDA) and monochorionic diamniotic twins (MCDA) in uncomplicated twin pregnancies (twin pregnancies without complications or comorbidities). The optimal gestational weeks of delivery for MCDA are 38 and 34–37^+6^ weeks, respectively, and the anesthetic modality for cesarean delivery is intravertebral anesthesia, including epidural, subarachnoid and spinal anesthesia [15]. Intraspinal anesthesia blocks the sympathetic nerves in the corresponding segment, reduces systemic vascular resistance, blood stays in the dilated vessels and reduces cardiac output [14, 15]. In this study, it was proposed to test the plane of anesthesia after completion of endotracheal anesthesia and to perform PVI recordings and IVC-CI measurements under US again, as several papers have shown that a reduction in PVI can be an early predictor of peripheral vasodilation after general anesthesia and epidural anesthesia, thus suggesting the onset of anesthetic block. Anesthetic drugs tend to exert their vasodilatory effects by increasing the vasodilatory threshold and decreasing the vasoconstriction threshold. Anesthesia causes redistribution of blood volume and body temperature, which in turn affects peripheral perfusion [15, 16]. The PVI can be used to continuously trace trends in waveform curves and can be extended in the future to conduct studies of pain management criteria in special populations, especially women who are unable to express pain intensity on their own or have poor communication.

The concept of enhanced recovery after surgery (ERAS) was first introduced by Danish scholar Kehlet in 1997, and ERAS brings shorter recovery time and fewer postoperative complications [17, 18]. Sound preoperative assessment and psychological intervention, appropriate preoperative sedation medication, shortened fasting time, fluid management and temperature intervention, multimodal analgesia, precise anesthesia, and prevention and control of perioperative complications comprise the basic requirements of ERAS for the anesthesia discipline in twin pregnancies [7, 18]. However, due to the lack of existing studies, few studies on the application of ERAS related to volume assessment in twin pregnancies have been seen in China. The dynamic predictive role of PVI in the prevention of supine hypotensive syndrome (SHS) has also been reported, but there are currently few studies on the application of perinatal volume assessment tools to accelerate the surgical recovery of women undergoing cesarean section. There is a lack of systematic studies on volume management in twin pregnancies, and there is no study on the use of PVI in combination with IVC-CI in the perinatal period for the moment.

In this study, due to the limitation of the number of cases and study time, a large sample and long period study was not conducted, pending the inclusion of more IVC-CI influencing factors, refinement of the regression model and improvement of *R*^2^, whose correlation and consistency are to be further observed. After subarachnoid administration, some patients may receive vasoactive drugs due to hypotension, which may affect the results of the study. To eliminate this effect, once a parturient develops dominant hypotension after intraspinal anesthesia, she will be excluded from this trial. We hope to study correlation between these two monitoring methods in parturients with vasoactive drugs in the future.

In conclusion, given the non-invasive and continuous advantages of PVI, combined with the role of IVC-CI in volume prediction, the combination of the two has correlation and consistency in cesarean delivery in twin pregnancies, and can improve sensitivity and specificity, making it a valuable predictor of cesarean delivery volume in twin pregnancies in clinical practice.

## Data Availability

All data generated or analyzed during this study are included in this published article.
